# Amylose Inter-Chain Entanglement and Inter-Chain Overlap Impact Rice Quality

**DOI:** 10.3390/foods11101516

**Published:** 2022-05-23

**Authors:** Changfeng Li, Yi Ji, Shaobo Zhang, Xiaoyan Yang, Robert G. Gilbert, Songnan Li, Enpeng Li

**Affiliations:** 1 Jiangsu Key Laboratory of Crop Genetics and Physiology, State Key Laboratory of Hybrid Rice, College of Agriculture, Yangzhou University, Yangzhou 225009, China; dx120170065@yzu.edu.cn (C.L.); mz120201195@yzu.edu.cn (Y.J.); mx120200716@yzu.edu.cn (X.Y.); b.gilbert@uq.edu.au (R.G.G.); lsnyz2020@yzu.edu.cn (S.L.); 2 Jiangsu Key Laboratory of Crop Genomics and Molecular Breeding, Jiangsu Co-Innovation Center for Modern Production Technology of Grain Crops, Yangzhou University, Yangzhou 225009, China; 3 Joint International Research Laboratory of Agriculture and Agri-Product Safety, Ministry of Education of China, Yangzhou University, Yangzhou 225009, China; 4 Center for Nutrition and Food Sciences, Queensland Alliance for Agriculture and Food Innovation, The University of Queensland, Brisbane, QLD 4072, Australia; shaobo.zhang@uq.net.au

**Keywords:** texture, amylose, fine structure, size-exclusion chromatography, entanglement

## Abstract

Retrogradation of cooked rice happens in two ways: one is by the formation of ordered structures, and the other is through intra- and inter-chain entanglement and inter-chain overlap, which in turn are affected by the amylose chain-length distribution. Both entanglement and overlap could affect rice texture. Here, four amylose samples were isolated from starch by precipitation from a dimethyl sulfoxide solution with butan-1-ol and isoamyl alcohol. Following enzymatic debranching, they were then characterized using size-exclusion chromatography. Amylose solutions (10%, *m*/*v*) were made by dissolving amylose in 90% (*v*/*v*) DMSO. Amylose gels (10%, *w*/*v*) were made by dissolving amylose powders into hot water, followed by cooling. The rigidity of the amylose gels and the structural order were measured using a texture analyzer and X-ray diffractometer, respectively. In the amylose solution, for a given mass of polymer in a fixed amount of solvent, the total occupied volume was reduced when the polymer molecular weight was smaller, resulting in less inter-chain overlap and a lower viscosity of the amylose solution. The overall mobility and diffusion of the molecules were inversely related to the square of the molecular weight until the gelation concentration. Thus, amylose gels in which amylose had a lower molecular weight had a greater chance to permeate into other molecules, which counterintuitively led to more inter-chain entanglement and more rigid amylose gels during retrogradation. This information could help rice breeders improve rice quality by using the molecular structure of starch as a guide.

## 1. Introduction

Starch is mainly made of amylose and amylopectin. Amylose is a linear α(1→4)-linked glucan with a small number of long-chain branches with α(1→6) linkages, and amylopectin is very highly branched, with relatively short branches. As one of the most important sources of human food energy, rice starch has been subjected to extensive studies, especially with regard to the processing of rice and the eating quality of rice products. The retrogradation of starch, which has a significant effect on the properties and on processing, occurs during processing/cooking and subsequent storage [1,2,3,4] and is influenced by rice composition [5,6,7].

Starch retrogradation occurs in two ways. One is the recrystallization of hydrated starch chains, which has been widely studied; for example, different amylose structures can form cells with different sizes and thicknesses in the starch gel network during retrogradation [8]. Another is by the amorphous single chains in an amylose gel [9] undergoing inter-chain overlap and inter-chain entanglement; the latter produces a three-dimensional polymer network, thereby contributing to starch retrogradation [9,10] and thus impacting rice texture. However, the effects of amylose chain length on amylose inter-chain overlap and inter-chain entanglement have not been examined in depth.

Amylose structure and its location in native starch granules affect starch recrystallization, overlap, and entanglement. The locations of amylose in starch granules are (1) the amorphous lamellae, (2) the amorphous growth rings, and (3) interspersed or crystallized with amylopectin molecules [11,12,13,14]. Rice amylose has a number-average degree of polymerization of ~920–1110 [15]. Amyloses are slightly branched, with an average of 2–5 chains per molecule [15,16]. The molar ratio and weight ratio of branched to linear rice amylose molecules have been reported to be 0.22:0.78 and 0.32:0.68, respectively, for the japonica rice Nipponbare [17]. Amylose molecules located in amorphous lamellae or with a small molecular size can leach out of the grain easily during gelatinization [18,19], which in turn influences starch gelation and recrystallization.

The recrystallization and inter- and intra-chain entanglement of amylose occur under different conditions. At high lipid concentrations, amylose–lipid complexes can be present in native starch or formed during starch gelatinization. The presence of lipids, either in the native plant or by the addition of exogenous lipids to starch-based food systems, generally slows down starch digestion [20,21] and retards retrogradation after food processing and/or storage [22,23]. At lower lipid concentrations, an amylose molecule can be involved in a double-helix conformation with the lipid, giving rise to competition with the formation of a single helix [24]. In neutral aqueous solution, amylose has no helical character, instead adopting a random coil conformation [25] (depending on the ionic strength). A uniform gel can be formed by the molecular entanglement of amylose at concentrations above the coil-overlap concentration, while precipitation can take place below this concentration, and a uniform gel will not form [10]. The crystalline packing perfection of these precipitates deposited from dilute aqueous solution diminishes with an increase in the average amylose chain length and yields an X-ray powder diffraction pattern consistent with that of the B-type crystalline form of starch [9,26]. Besides recrystallization and entanglement, another molecular interaction, inter-chain overlap, is involved in amylose gelation. The critical concentration for gelation is lower than that for entanglement [9].

Amylose content is important for the elasticity of freshly retrograded starch dispersions and for the hardness of freshly cooked rice [27,28,29]. It also can lead to a high proportion of slowly digestible starch and resistant starch and thereby lower the glucose and insulin response. In addition, the amylose and amylopectin chain-length distributions (CLDs) are also major factors impacting the digestibility of retrograded starch [8].

Most previous studies of retrogradation focused only on recrystallization. In the present work, the effect of the amylose chain-length distribution on its entanglement and overlap are examined, seemingly for the first time (although amylose entanglement has been examined for dependence on the average chain length [30]).

Generally, amylose entanglement and overlap cannot be examined directly in whole rice or in rice flour, as amylopectin is also present in significant amounts. Amylose isolation is one way to solve this issue. Once the amylose is isolated, the retrogradation could be different from that of amylose in rice flour because the contributions from the formation of ordered structures might change. Amylose entanglement and overlap, including the effects of chain-length distributions, will still be present in isolated amylose, and thus knowledge of the effects of chain length on the chain entanglement and overlap in isolated amylose systems will help the understanding of what happens in whole rice.

In this work, four amylose samples with different fine structures were used to explore fine-structure effects on retrogradation and potential effects on rice sensory characteristics arising from chain entanglement and overlap. This understanding could supply information for breeding rice for special purposes. Extensive studies have shown which genes control the chain-length distributions and relative amounts of amylopectin and amylose in rice. Therefore, based on amylose entanglement (using appropriate chain-length distributions and amylose content), rice starch with a specific amylose structure could be developed and used in electrospinning for potential pharmaceutical applications for which an appropriate molecular entanglement is a prerequisite [31].

## 2. Materials and Methods

### 2.1. Materials

Three rice varieties (XiangGeng111, SanLuZhan, and Taizhou0206) with different amylose contents were sourced from a field trial grown at HuaiSi, YangZhou City, Jiangsu Province, China, in the summer season of 2018. Paddy rice was shelled using a laboratory-model rubber roller sheller (Satake, Tokyo, Japan) and polished into white rice with a laboratory-model rice polisher (Pearlest, Kett, Tokyo, Japan). Koshihikari (JA-RICE, Itabashi Trading Co., Ltd., Tokyo, Japan) white rice was bought online (Tmall Mart). White rice was ground and passed through a 100-mesh sieve to obtain fine flour. Pancreatin (Sigma P1750, from porcine pancreas, Sigma, St. Louis, MO, USA), amyloglucosidase (E-AMGDF, Megazyme, Wicklow, Leinster, Ireland), and 8-aminopyrene-1,3,6, trisulfonic acid (ATPS-M, Beckman, Brea, CA, USA) were obtained. Protease from *streptomyces griseus* (type XIV) was purchased from Sigma-Aldrich Pty. Ltd. (Castle Hill, NSW, Australia); corn starch (K-TSTA) was from Megazyme, Ireland; and amylose (Sigma A0512, from potato, for the calibration of amylose content determination by iodine staining) was from Sigma-Aldrich (Milwaukee, WI, USA). All other chemicals were of analytical grade.

### 2.2. Starch and Amylose Isolation

The samples were pre-treated with protease to remove proteins using a published procedure [31] with some modifications. Raw rice flour (30 g) was incubated with protease (0.9 U/mL) overnight at 37 °C. The slurry was centrifuged at 4000× *g* for 10 min. The sediment (starch) was washed with distilled water and then with absolute ethanol three times after removing the yellowish layer of protein and then dried in a convection oven at 40 °C for 48 h. The dried starch was stored in a desiccator for further use.

Amylose isolation was conducted as described previously [32] with some modifications. Purified starch (10 g) was dispersed in 200 mL of 90% (*v*/*v*) DMSO (diemthyl sulfoxide) in a boiling-water bath with constant stirring for 3 h. Four volumes of ethanol were added, and the mixture was then centrifuged at 4000× *g* for 15 min. The supernatant was discarded, and the pellet was washed twice with absolute ethanol and once with acetone, then dried in a convection oven at 50 °C for 24 h. The resulting dried starch was dissolved in 70 mL of 90% (*v*/*v*) DMSO. Seven volumes (490 mL) of an aqueous mixture of 6% butan-1-ol and 6% isoamyl alcohol (3-methyl butan-1-ol) were added and incubated with constant stirring in a 95 °C water bath for 1 h. After cooling to room temperature, the mixture was centrifuged at 10^4^× *g* for 15 min at 4 °C. The supernatant was discarded, and the precipitate was resuspended in water to bring the starch concentration to ~1–2%. Isoamyl alcohol and butan-1-ol were added to repeat the precipitation. After three such precipitations, the precipitate was re-dispersed by stirring in 70 mL of 90% (*v*/*v*) DMSO and then mixed with 7 × the volume of 6% butan-1-ol. This mixture was heated and centrifuged as described above. The final precipitate (amylose fraction) was dispersed in 50 mL of 90% DMSO, precipitated with 6 × the volume of ethanol, washed, and dried as described above. The dried sample was stored in a desiccator for further use. When isoamyl alcohol was used, the combination of butan-1-ol and isoamyl alcohol would remove contaminating non-precipitated material (amylopectin) in the precipitation procedure. After three precipitations by butan-1-ol and isoamyl alcohol, the relative purity of the isolated fractions was increased, but there was also intermediate material [32,33]. When the procedure was followed by three or more precipitations with butan-1-ol alone to remove the intermediate material, amylose samples with higher purities were obtained [34]. For the amylose sample from XiangGeng111, only three precipitations were conducted, with butan-1-ol alone. Different solvents were used here to obtain amylose samples of different purities to see the effects of the presence of amylopectin on the properties of amylose gel.

### 2.3. Collection of Leached Starch during Rice Cooking

Leached starch was collected as described elsewhere [19]. Briefly, rice grains (20 g) were washed three times with distilled water. The washed rice was placed in a beaker with distilled water at a rice-to-water ratio of 1:1.4 and covered with a filter paper, followed by cooking in a steam cooker (KuaiLeYiDing, DZG-A21, Guangdong, China) for 45 min. The cooked rice was rinsed twice with 100 mL of hot deionized water with gentle stirring for 10 min, followed by filtering through a 100-mesh sieve. The resulting solution was frozen immediately in liquid nitrogen and lyophilized with a freeze dryer (BTP, SP Scientific, Gardiner, NY, USA) to obtain the leached materials.

### 2.4. Molecular Size Distribution of Fully Branched Parent Starch, Leached Starch, and Isolated Amylose

The size distributions of the fully branched parent starch, leached starch, and isolated amylose were obtained using an LC-20AD Shimadzu SEC system (Kyoto, Japan) equipped with a Shimadzu RID-10A differential refractive index detector (Shimadzu Corporation, Kyoto, Japan), as described elsewhere [28]. Analytical SEC columns (GRAM 30 and GRAM 3000, Polymer Standard Service, Mannheim, Germany) were used to obtain the size distributions of the fully branched samples. The eluent was a DMSO/LiBr solution (0.5% *w*/*w*) with a flow rate of 0.3 mL/min in a column oven set at 80 °C. The whole separation period was about 2 h. SEC separates by molecular size, specifically the SEC hydrodynamic radius, *R*_h_, not by molecular weight. Briefly, when molecules are injected into the SEC column and are carried by the solvent along the column packed with porous particles, smaller molecules will have a more tortuous path through the column because they can enter smaller pores than can larger particles and, hence, will elute more slowly [33]. For any branched polymer such as starch, there is no unique relation between molecular size and molecular weight. The SEC weight distribution (*w*(log*R*_h_)) for fully branched samples was plotted against the hydrodynamic radius (*R*_h_), which was converted from elution time using universal calibration from standards with known *R*_h_ values [34].

### 2.5. Starch Debranching and Measuring of the CLD of Debranched Parent Starch, Leached Starch, and Isolated Amylose by Size-Exclusion Chromatography

The starches were enzymatically debranched with isoamylase, an enzyme that exclusively cleaves α-1,6-glycosidic bonds at the branch point [35]. For debranched starch, the resulting linear molecules have a unique relationship between the molecular weight and molecular size. The debranched distributions can be presented both in terms of the degree of polymerization (DP) and the hydrodynamic radius (*R*_h_), using the Mark–Houwink relationship [36]. The size distributions of the debranched parent starch, leached starch, and isolated amylose were obtained using an LC-20AD Shimadzu SEC system (Kyoto, Japan) equipped with a Shimadzu RID-10A differential refractive index detector (Shimadzu Corporation, Kyoto, Japan). Analytical SEC columns (GRAM 100 and GRAM 1000, Polymer Standard Service, Mannheim, Germany) were used to obtain the size distributions of the debranched samples. The eluent was a DMSO/LiBr solution (0.5% *w*/*w*) with a flow rate of 0.6 mL/min in a column oven set at 80 °C. The whole separation period was about 60 min. The SEC weight distribution, *w*(log*X*), was plotted against DP *(X*) using a universal calibration from the standards with known molecular weights [37].

### 2.6. Fitting Amylopectin CLD with a Biosynthesis Model

In order to show the fine structure differences in parent starch, leached starch, and isolated amylose, the amylose CLDs were fitted by the biosynthesis-based model of Nada, Zou, Li, et al. [38]. This model is based on the fact that the CLDs show a number of distinguishable features, e.g., maxima or shoulders, each of which is assumed to be predominantly, but not exclusively, synthesized by an enzyme set comprising a starch synthase, a starch-debranching enzyme, and one or two starch branching enzymes. The models reduce to sets of parameters, *β* and *h*, for each enzyme set, representing the ratios of the rate of chain stoppage over that of chain growth in enzyme sets and the amount of amylose/amylopectin resulting from that enzyme set, respectively. The amylose content was obtained directly from the debranched CLD [39] and was also measured by iodine staining [40].

### 2.7. Dynamic Rheological Properties of Amylose Sol

An amylose sol (10%, *m*/*v*) was prepared by dissolving amylose in 90% (*v*/*v*) DMSO at 80 °C overnight. The rheological behavior of the sol was measured with a Discovery Hybrid Rheometer (HR1, TA Instruments, New Castle, DE, USA) equipped with a parallel plate geometry (40 mm diameter). The amylose suspension was loaded between the parallel plates in the mechanical spectrometer, and the upper plate was lowered to leave a gap of 500 μm. The excess suspension around the plate was gently scraped off, and the edge of the sample was covered with silicone oil to prevent water evaporation. The linear viscoelastic region of the sample was determined with a strain sweep (0.01~100%) at a fixed frequency of 1 Hz. An oscillatory frequency sweep over a range of 0.1 to 100 rad/s at 37 °C was then applied [41,42].

The mechanical spectra were parameterized with a power-law model to quantify the differences among the samples [41,42]:

*G*′ = *K*′ *f n*′

*G″ =K″ f n″*

Here, *K*′ and *K*″ are the coefficients that represent the storage and loss modulus, respectively, at 1 Hz, and *n*′ and *n*″ are the slopes of the curve in the log-log plot of *G*′ and *G*″ versus frequency *f*. All measurements were performed in triplicate.

### 2.8. Preparation of Amylose Gel

First, 300 mg of the amylose powder obtained as above was dissolved in 5 mL of DMSO at 80 °C overnight. The amylose was then precipitated by adding six times the volume of ethanol and centrifuging at 4000× *g* for 10 min. The supernatant was discarded, and the precipitate was centrifuged again to remove the residual ethanol. These steps facilitated the dissolution of amylose in water. The final precipitate was dissolved in 3 mL of warm water and incubated for 30 min in a 95 °C water bath. The hot aqueous amylose sol (10%) was transferred into plastic circular molds (24 disks, 15.6 mm diameter and 18 mm height) and spread out evenly. The sample was allowed to cool for 1 h at room temperature and then stored at 4 °C for 12 h to stabilize the gels before texture analysis.

### 2.9. Texture Profile Analysis (TPA) of Amylose Gel and Cooked Rice

Textural attributes of the gels were characterized using a Texture Analyzer (TA.XT plus, Stable Micro Systems Ltd., Godalming, UK) equipped with a P36R cylindrical probe using a two-cycle, force-versus-distance compression program. The settings were as follows: pre-test speed, 1 mm/s; test speed, 0.8 mm/s; post-test speed, 1 mm/s; distance 3 mm; time 5 s; trigger force, 5 N.

For the cooked rice texture properties, rice was cooked as described in Section 2.3 and was then allowed to cool for 10 min under ambient conditions. After this, 1 g samples of cooked rice, excluding both material in the top layer and that adhering to the sides and bottom of the container, were placed on the sample plate of the texture profile `analyzer with single grain thickness. The settings were as follows: pre-test speed, 1 mm/s; test speed, 0.8 mm/s; post-test speed, 1 mm/s; strain 70%; time 5 s; trigger Force, 30 N. Quintuplicate measurements were conducted to minimize the error at room temperature.

### 2.10. X-ray Diffraction (XRD)

The X-ray diffraction patterns of both the freeze-dried amylose and amylose with 10% moisture after exposure to atmosphere at room temperature were analyzed with an X-ray diffractometer (Bruker, D8 Advance, Karlsruhe, Baden-Wurttemberg, Germany), as described elsewhere [43].

### 2.11. Digestion Analysis and Model Fitting

In vitro amylose digestibility was measured as described previously [37]. Briefly, samples (~100 mg) were mixed with 10 mL of enzyme solution that was pre-warmed to 37 °C (0.5 mg of pancreatin and 100 μL of amyloglucosidase with 9.9 mL of sodium acetate buffer (0.2 M, pH 6.0)) for digestion. A 100 μL aliquot was collected and mixed with 0.9 mL of absolute ethanol after 720 min of incubation. The glucose concentrations were determined by a UV-1700 Pharma Spectrophotometer at 510 nm after incubation with a glucose oxidase/peroxidase reagent (GOPOD, Megazyme). The data, comprising the concentration of released glucose as a function of time, were fitted using the logarithm of slope (LoS) method and the non-linear least-squares fitting method (NLLS) [44]. The LoS method shows if one has a single or multiple first-order rate steps during digestion. NLLS fitting then gives accurate coefficients of the digestion rate coefficient, *k,* and the long-time fraction of undigested starch, *C*_res_, in each of the single-step regions suggested by the LoS method, as described previously [37].

### 2.12. Statistical Analysis

For each measurement, data were expressed as means ± standard deviations (SD) of at least two runs. A one-way ANOVA was carried out by Duncan’s multiple-range test using IBM SPSS software (IBM SPSS 25.0, New York, NY, USA) to analyze the significant differences between samples. Correlations among the physicochemical properties and structural parameters were assessed with Pearson’s correlation analysis, also using IBM SPSS. A *p* value ≤ 0.05 was regarded as significant throughout the study.

## 3. Results and Discussion

### 3.1. Molecular Size Distributions of Parent Starch, Leached Starch, and Isolated Amylose

**Figure 1:**
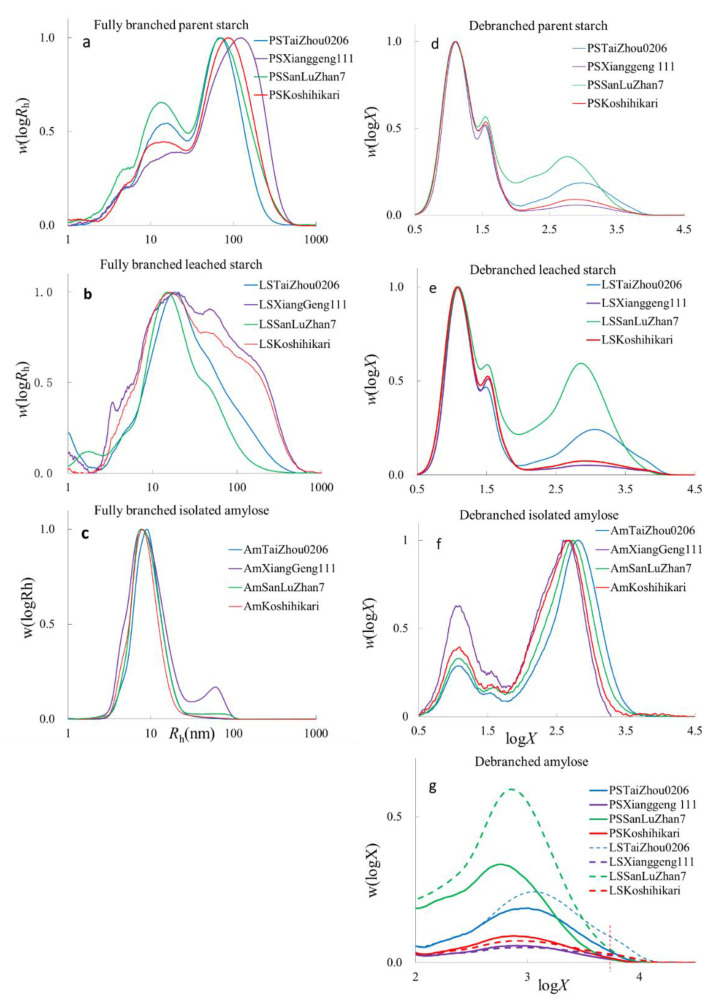
SEC weight distributions of branched and debranched starch molecules. (**a**,**d**): parent starches; (**b**,**e**): leached starches; (**c**,**f**): isolated amyloses. (**a**–**c**): plots of *w*(log*R*_h_) for fully branched samples plotted against *R*_h_. (**d**–**f**): plots of *w*(log*X*) for debranched samples plotted against DP (*X*); (**g**): enlarged representation of the amylose portion for parent starch and leached starch. The vertical broken line in (**g**) indicates the extra peak in leached starches, which is difficult to see in the parent starches.

The SEC weight distributions *w*(log*R*_h_), being the weight distributions of molecules as a function of (logarithmic) size, which are the SEC hydrodynamic radii, *R*_h_, of whole starch molecules, parent starch, leached starch, and isolated amylose, are shown in Figure 1. As is commonly seen, the fully branched distributions of parent starch show two peaks (Figure 1a), amylose (*R*_h_ < 30 nm) and amylopectin (*R*_h_ ≥ 30 nm). An additional peak at *R*_h_~3–4 nm is thought to be residual proteins, probably because of the incomplete hydrolysis by protease during starch isolation [31]. Fully branched leached starches have amylose and amylopectin peaks, although they are not as distinct as those of parent starch (Figure 1b). All fully branched isolated amyloses have an amylose peak, while amylose from XiangGeng111 (Am_XiangGeng111_) has a wide range of molecular sizes with residual amylopectin peaks because the amylose separation procedure is not 100% effective (Figure 1c). Given that Am_XiangGeng111_ was isolated with butan-1-ol alone and the other three samples were isolated using isoamyl alcohol and butan-1-ol, it is seen that amylose enrichment using butanol alone can yield samples with increased amylose but not pure amylose. The use of isoamyl alcohol and butan-1-ol for amylose isolation was more efficient than butan-1-ol alone. In fully branched parent starch (PS) samples with lower amylose contents, PS_XiangGeng111_ and PS_Ksohihikari_ had higher $\bar{R}$_h,Ap_ and $\bar{R}$_h,Whole starch_, the last two quantities being average sizes. Samples with higher amylose contents, PS_SanLuzZhan7_ and PS_TaiZhou0206_, had small average sizes of amylopectin, $\bar{R}$_h,Ap_, and of whole starch, $\bar{R}$_h,Wholestarch_ (Table S1). This was also found in the leached starch. Leached starches (LS) from cooked Koshihikari (LS_Koshihikari_) and XiangGeng111 (LS_XiangGeng111_) rice contained more amylopectin than those of Taizhou0206 (LS_Taizhou0206_) and SanLuZhan7 (LS_SanLuZhan7_) (Figure 1a,b). As expected, the values of $\bar{R}$_h,Whole starch_ of the leached starches were smaller than those of parent starch, as seen elsewhere [28]. The $\bar{R}$_h,Am_ of leached starch and isolated amylose from SanLuZhan7 and TaiZhou0206 were larger than those of the other two. The $\bar{R}$_h,Am_ of isolated amylose was smaller than those of the parent and leached starches. It is speculated that not all of the amylose was extracted using the above method.

The CLDs of all samples showed typical amylopectin peaks, as can be observed in Figure 1d–f. For the parent starch, PS_SanLuZhan7_ and PS_TaiZhou0206_ had higher amylose contents than PS_XiangGeng111_ and PS_Koshihikari_. In the leached starch, the amylose contents of samples LS_SanLuZhan7_ and LS_TaiZhou0206_ were higher than in the parent starch, which means a higher relative amount of amylose was leached out of both types of rice during cooking (Figure 1g and Table S2). The amylose contents of LS_XiangGeng111_ and LS_Koshihikari_ were the same as those of their parent starches, probably due to the low amylose content in the parent starches (Figure 1). It is worth mentioning that only two peaks exist in the parent starches in the amylose range, but three peaks can be seen in the leached starches (Figure 1g). The extra peak is at log*X*~3.8, indicating that more long-chain amylose molecules are leached out during cooking. This is different from a previous report [45] stating that leached amylose has a much narrower DP range than native amylose. In that earlier work, an unintentional loss of amylose may have happened during the deproteinization procedure because of the solubility of leached amylose in the protease buffer, which was discarded in their procedure; this loss might have led to an inaccuracy in CLDs for leached starches if the loss were biased towards amylose molecules of different sizes. In our work, the supernatant, which contained amylose and could be stained by iodine solution after deproteinization and centrifugation, was retained and characterized to better understand the intrinsic structural characteristics of leached amylose. As seen in Figure 1e, the leachate contained more long-chain amylose, not just short amylose, and is thus expected to be more representative of the characteristics of amylose in the native rice.

Here, all isolated amyloses were found to be contaminated with amylopectin (Figure 1f), presumably containing extra-long chains. This agrees with reports that chemical precipitation can yield high amylose fractions, but the complete separation of pure fractions was not achieved [33]. The amylose contents calculated from the SEC data for Am_TaiZhou0206_, Am_XiangGeng111_, Am_SanLuZhan7_, and Am_Koshihikari_ were 80.2%, 71.4%, 77.6%, and 76.1%, respectively. The amylose contents measured by the colorimetric method for Am_TaiZhou0206_, Am_SanLuZhan7_, and Am_Koshihikar_ were about 100% (Table S3), higher than those calculated using the SEC data. The reasons for this have been pointed out [39]: different methods for measuring “amylose content” actually measure different properties. These methods would not necessarily give the same result: e.g., the property being measured by the SEC data is dependent on the amylose CLD, and that measured by the colorimetric method is based on amylose CLD and the chain’s binding abilities to iodine. The amylose content by the colorimetric method for Am_XiangGeng111_ was 64.9% (only butan-1-ol was used for this sample). The average amylose chain lengths in isolated amylose samples were shorter than those in their parent starches (Table S2), which may be caused by the methods used for amylose isolation. The hydrodynamic radii of amylopectin ($\bar{R}$_h,de,Ap_) in isolated samples were higher than those in their parent starches, meaning there were longer amylopectin chains in the isolated samples. This result indicates that the extra-long amylopectin could be precipitated by butan-1-ol. The differences in amylopectin content among the isolated amyloses were similar, except for Am_XiangGeng111_; the main difference between Am_TaiZhou0206_, Am_SanLuZhan7_, and Am_Koshihikar_ was that the CLD of the amylose component (amylose suspensions and gels) for Am_TaiZhou0206_, Am_SanLuZhan7_ and Am_Koshihikar_ were caused by their CLDs.

### 3.2. Model Fitting Parameters for Debranched Starch

The amylose components from parent and leached starches were fitted to a biosynthesis-based model for native starch. This model is based on the biosynthetic processes controlling the CLD in native starch and, of course, is not directly applicable to the present systems, as they do not take into account the degradation process used to make these samples. However, it may be possible to parameterize these CLDs by fitting the biosynthesis-based models to the present CLD because these models effectively provide a flexible functional form. Indeed, it was found that most of these data can be adequately fitted by this empirical method (which is fortunate, as there was no a priori reason that this empirical parameterization should work), except for the CLDs of all isolated amyloses. Examples of amylose CLD empirical fitting (including the one where the method does not work) are given in Supplementary Information (Figure S1), showing the CLDs and model fitting of amylose and amylopectin from rice TaiZhou0206 as examples. The biosynthesis-based empirical fitting parameters of amylose are summarized in Table 1. Higher *β* values mean shorter amylose chains, and smaller *h* values mean a smaller contribution of amylose in that region to the total amount of amylose. As seen in Figure 1g, only two peaks were observed in the parent starch, with three in the leached starch (columns with better separation than those used here might resolve more features, but that would not assist to fulfill the aim of the present paper). Thus, the parameters *β*_AmP,i_, *h*_AmP,i_, *β*_AmP,ii_, and *h*_AmP,ii_ were obtained for parent starch and *β*_AmL,i_, *h*_AmL,i_, *β*_AmL,ii_, *h*_AmL,ii_, *β*_AmL,iii_, and *h*_AmL,iii_ were obtained for leached starches. Details are shown in the Supplementary Materials (Figure S1), showing the CLDs and model fitting of amylose and amylopectin from rice TaiZhou0206 as examples. It is seen that the second peaks were always dominant in whole amylose. For the parent starches, the values of *β*_AmP*,*ii_ of PS_SanLuZhan7_ and PS_TaiZhou0206_ were, respectively, significantly higher and lower than those of PS_XiangGeng111_ and PS_Koshihikari_ (Table 1), indicating shorter medium and long amylose chains in PS_SanLuZhan7_ than other types of rice and the longer medium and long amylose chains of PS_TaiZhou 0206_ than the others. This characteristic was also seen in the leached starches. For example, the values of *β*_AmL,ii_ and *β*_AmL,iii_ for PS_SanLuZhan7_ were the highest, indicating that branches of extra-long amylose in leached starch from rice SanLuZhan7 were shorter on average than those in leached starch from Xianggeng111, Koshihikari, and Taizhou0206. The existence of the third peak also reveals that amylose with long branches leaches out more easily. An explanation is that some of the amylose molecules are located in the amorphous lamellae, not in the amorphous growth rings, and are not associated with amylopectin in the form of crystals [18]. They are, thus, more easily leached out during gelatinization.

### 3.3. Order in Amylose Gel

**Figure 2:**
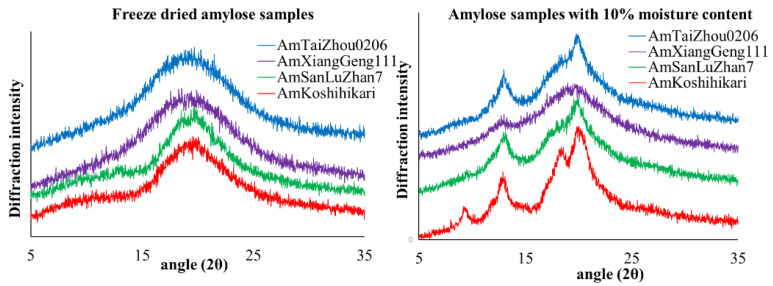
X-ray diffraction patterns for freeze dried amylose samples (**left figure**) and amylose samples with 10% moisture content (**right figure**).

X-ray diffraction analyses of the freeze-dried amylose gels showed a typical broad amorphous halo (Figure 2), indicating low crystallinity. Thus, the formation of the amylose gels is attributed to intra- and inter-chain entanglement instead of amylose crystallinity. This is consistent with previous work [46,47,48], indicating that amylose inter-chain entanglement occurs at the rapid stage of retrogradation, while the entanglement of ordered or crystalline amylopectin and the interaction between amylose and amylopectin take place gradually and in the amylose gel. Here, the aging time was 12 h, which may not be long enough for crystallization in the amylose gel. However, a significant crystallinity for isolated amylose powders with moisture contents of 10% was detected (Figure 2 and Table 2). Am_Koshihikari_ had the highest degree of crystallinity, while Am_SanLuZhan_ had the lowest. Given that amylose was well-dispersed in excess water during the gelation process, a possible reason is that the amylose has freer access to water at the higher temperature**s** and that intra- and inter-chain entanglement dominate the retrogradation. Moreover, the access to water is limited at room temperature (when amylose cannot completely disperse in water in the present system), so crystallization can take place.

### 3.4. Dynamic Rheological Properties of Amylose

**Figure 3:**
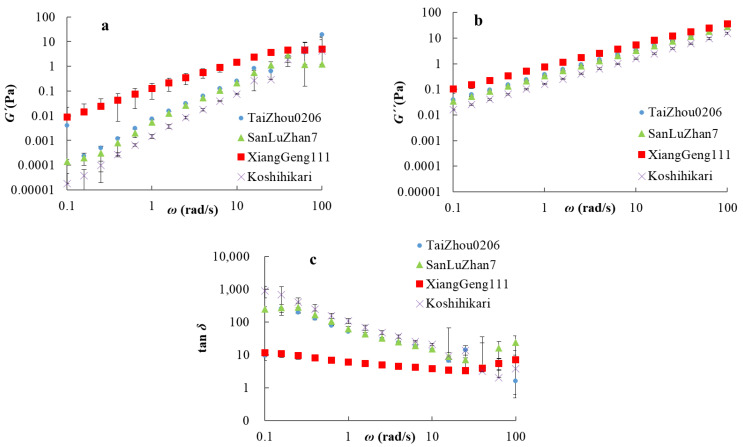
Dynamic viscoelasticity as a function of frequency, ω, for amylose solutions: (**a**): dynamic storage moduli (*G′*); (**b**): dynamic loss moduli (*G*″); (**c**): loss tangent (tan δ).

Dynamic rheology was used to help make inferences about the intra- and inter-chain entanglement and inter-chain overlap of amylose suspensions. The dynamical viscoelastic properties of amylose were measured using an oscillatory frequency sweep. *G′* represents the elasticity of the sample, *G*″ the viscosity of the sample, and tan *δ* reflects the solid-like or liquid-like properties of the sample [41]. As shown in Figure 3, the values of storage and loss moduli of all samples increased with the oscillatory frequency, *ω.* An increase in *G′* with *ω* was commonly observed in amylose suspensions [49,50]. The value of tan *δ* decreased in the low frequency range (0.1–25 rad/s), increased at high frequencies (25–100 rad/s), and was always >1, indicating predominantly viscous behavior rather than elastic; the amylose suspensions had a typically viscous fluid behavior [50]. The mechanical spectra of *G′*, *G*″ and tan *δ* were similar in Am_Taizhou0206_, Am_SanLuZhan7_, and Am_Koshihikari_; that of Am_XiangGeng111_ was noticeably different from amylose suspensions Am_Taizhou0206_, Am_SanLuZhan7_, and Am_Koshihikari_, probably because of amylopectin contamination. This agrees with reports that the *G′* and *G*″ values of waxy rice starch are higher than those of non-waxy rice starch [49].

In order to quantify the differences among those samples, the power-law model was applied to fit the dynamic viscoelasticity data of the mechanical spectra (R^2^ > 0.99). The parameters obtained for log*K′,* log*K*″, *n′,* and *n*″ are listed in Table 3. In comparison with the other three samples, the values of log*K′* and log*K*″ of Am_XiangGeng111_ were significantly higher. For Am_Koshihikari_, the values of log*K′* and log*K*″ were significantly lower than those of the other samples. The results showed that Am_Koshhikari_, with a smaller molecular size and shorter branches, had the lowest elasticity and viscosity, while Am_XiangGeng111_ with larger molecular size and longer chains has the highest elasticity and viscosity, as seen previously [46]. The differences in the elasticity and viscosity are ascribed to the overlap between amylose molecules because the only structural differences among these samples were the molecular size and chain-length distribution. An explanation is that amylose with a higher molecular weight has a higher radius of gyration, leading to more inter-chain overlap in the suspension (see the proof of this in the Supplementary Information), which restricts the free movement of the molecules, causing a greater increase in viscosity.

### 3.5. Texture of Cooked Rice and Amylose Gel

The hardness and stickiness of cooked rice is shown in Table 4. Significant differences in the hardness of cooked rice were observed, except between TaiZhou0206 and SanLuZhan7. Both the hardness and stickiness of the Koshihikari samples were higher than those of XiangGeng111, which goes against the perception that harder rice goes with low stickiness [51]. Considering the higher amylose content (Table S2) and the smaller molecular size of Koshihikari starch (Table S1) compared to XiangGeng111, the harder texture of Koshihikari rice could be caused by the higher amylose content because amylose CLD as well as amylose content have been considered as the determinants for textural properties such as those studied here [52]. It is suggested that the stickier texture is caused by the smaller molecular size of Koshihikari starch, which leaches out more easily during cooking compared to XiangGeng111, consistent with a report that higher amounts of leached materials make rice stickier [19].

The gel hardness of isolated amyloses is shown in Table 4. The stickiness of amylose gel measured by TPA is less accurate than that of cooked-rice stickiness. Amylose gel is a homogeneous glue-like translucent material, and leaching does not occur during gel formation or retrogradation. Thus, the stickiness of amylose gel does not reflect cooked-rice stickiness. The hardness of amylose gels in descending order were Am_Koshihikari_, Am_SanLuZhan7_, Am_TaiZhou0206_, and Am_XiangGeng111_, and the differences were significant. The gel of Am_XiangGeng111_ had the lowest values of hardness, probably because of the contamination with amylopectin (Figure 1). This is consistent with a previous study that stated that mixtures of amylose and amylopectin do not form gels, even weak gels, during storage [53]. Among the other three, Am_Koshihikari_, with the smallest molecular size and shortest chains, formed the most rigid gel, and Am_TaiZhou0206_, with the largest molecular size and longest chains, was the least rigid. This indicates that amylose molecules with smaller sizes and shorter chains are more likely to form a harder gel. XRD results showed a typical “amorphous” halo of retrograded amylose gel (the same as that of freeze-dried amylose), implying the hardness of the amylose gel is caused by inter-chain entanglement. This is consistent with a report [54] that higher molecular weight amylose fractions produce less rigid gels at a given concentration. Here, the conditions for gel formation were (1) the concentration of amylose solutions was 10% (*w*/*v*), which is higher than the critical concentration for the formation of an amylose gel (~1%, *w*/*v*) and molecular entanglement (less than 7.2%, *w*/*v*) [30], and (2) the overall mobility and diffusion coefficients of the chains were proportional to *M*^−2^ [55]. It is speculated that, for a given mass of polymer in a fixed amount of water, smaller molecules (or chains) have a greater chance to permeate into other molecules than bigger ones because of their higher mobility and diffusion, providing a greater opportunity for inter-chain entanglement in amylose solution and leading to a more rigid texture after gel retrogradation. This is consistent with a report that shorter amylose chains result in harder cooked rice compared to long amylose chains [56].

### 3.6. Amylose Digestion Kinetics

The LOS plots of the enzymatic digestion of amylose solutions (using isolated amylose) here showed an initial fast digestion step, followed by a slow second step. Both regions were fitted using the NLLS method (Table S4). (1) The digestion rate coefficient (*k*) in the first digestion step of Am_Koshihikari_ was significantly lower than those of the others. Am_XiangGeng111_ had the highest digestion rate among these samples, which can be explained by starch with more amylopectin being easier to hydrolyze to glucose [57,58,59,60] because having more end-groups of amylopectin means a greater opportunity for enzymatic hydrolysis. Sample Am_Koshihikari_ had the largest fraction of residual undigested starch, *C*_res_, at long times, while sample Am_XiangGeng111_ had the smallest (Table S4). The reason is that when amylose powder was added to the in vitro digestion system, shorter amylose had a higher extent of inter-chain entanglement at the periphery of the powdery particles where amylose could contact water to form a hard gel, which decreased the susceptibility of amylose molecules to enzymatic hydrolysis. (2) In the second digestion step, the overall rates were significantly lower than those in the first step, although statistically significant differences in digestion rates were only found in samples Am_Koshihikari_ and Am_XiangGeng111_. This may have been caused by the presence of amylopectin in Am_XiangGeng111_ and/or the difference in amylose structure. The values of *C*_res_ (the long-time undigested fraction) in the second step for each sample were less than those in the first step, and significant differences existed among all the samples (Table S4). It is seen here (Figure 1 and Table S4) that the digestion rate of amylose was significantly affected by the itsamylose CLD, and amyloses with smaller molecular sizes and shorter branches digested more slowly because of the inter-chain entanglement among amylose molecules, as in the first digestion step.

## 4. Conclusions

Four isolated rice amyloses were used to reveal that, for a given mass of polymer in a fixed amount of solvent, a smaller molecular weight leads to (1) a lesser extent of inter-chain overlap in the suspension, which increases the mobility of the molecules, causing a greater decrease in viscosity; (2) a greater chance of inter-chain entanglement makes amylose gels more rigid; and (3) the greater chance of inter-chain entanglement retards amylose digestion by hindering enzyme access.

This work provides a new understanding about amylose retrogradation, which could prove to be useful information for the breeding and selection of rice for special purposes.

## Figures and Tables

**Table 1:** Parameter values from fitting the biosynthesis-based model to the amylose CLDs for parent starch and leached starch.

Sample	Model Fitting Parameters for Parent Starch				Model Fitting Parameters for Leached Starch					
	*β*_AmP,i_/10^−3^	*h*_AmP,i_/10^−3^	*β*_AmP*,*ii_/10^−3^	*h*_AmP,ii_/10^−3^	*β*_AmL,i_/10^−3^	*h*_AmL,i_/10^−3^	*β*_AmL*,*ii_/10^−3^	*h*_AmL,ii_/10^−3^	*β*_AmL,iii_/10^−3^	*h*_AmL,iii_/10^−3^
TaiZhou 0206	14.4 ± 4.5 ^ab^	20.2 ± 3.9 ^bc^	1.8 ± 0.0 ^a^	148.4 ± 2.4 ^d^	18.0 ± 0.6 ^a^	31.1 ± 3.1 ^b^	2.0 ± 0.1 ^a^	171.1 ± 9.7 ^c^	0.6 ± 0.0 ^b^	86.7 ± 0.5 ^b^
XiangGeng111	13.6 ± 2.5 ^ab^	8.1 ± 1.4 ^ab^	2.1 ± 0.1 ^b^	48.2 ± 2.5 ^b^	11.8 ± 0.1 ^a^	13.2 ± 1.0 ^a^	2.1 ± 0.0 ^a^	36.0 ± 0.3 ^a^	0.5 ± 0.0 ^a^	20.6 ± 1.3 ^a^
SanLuZhan7	16.7 ± 0.9 ^ab^	101.2 ± 2.5 ^d^	3.0 ± 0.0 ^e^	264.6 ± 1.2 ^f^	24.4 ± 0.9 ^a^	148.3 ± 12.9 ^c^	2.7 ± 0.0 ^c^	437.5 ± 4.8 ^d^	0.8 ± 0.0 ^c^	85.9 ± 1.4 ^b^
Koshihikari	8.9 ± 5.4 ^a^	3.7 ± 1.9 ^a^	2.1 ± 0.1 ^b^	71.2 ± 0.1 ^c^	18.6 ± 1.6 ^a^	11.4 ± 0.3 ^a^	2.5 ± 0.1 ^b^	52.8 ± 0.4 ^b^	0.5 ± 0.0 ^ab^	27.3 ± 0.1 ^a^

The values are means ± SD, calculated from duplicate measurements; values with different letters for the same parameter are significantly different at *p* < 0.05.

**Table 2:** The degree of crystallinity in amylose samples with 10% moisture content.

Sample	Degree of Crystallinity (%)
Am_TaiZhou0206_	14.0 ± 0.2 ^b^
Am_XiangGeng111_	7.1 ± 0.1 ^a^
Am_SanLuZhan7_	16.3 ± 0.3 ^b^
Am_Koshihikari_	24.4 ± 2.1 ^c^

The values are means ± SD, calculated from duplicate measurements; values with different letters in the same column are statistically different at *p* < 0.05.

**Table 3:** Power-law model fitting parameters.

Sample	log*K′*/10^−2^	*n′*	log*K*″10^−2^	*n*″
Am_Taizhou0206_	−162.0 ± 4.1 ^b^	2.2 ± 0.0 ^b^	−97.4 ± 0.0 ^b^	0.4 ± 0.0 ^b^
Am_XiangGeng111_	−107.3 ± 0.4 ^a^	0.9 ± 0.1 ^a^	−84.2 ± 1.5 ^a^	0.1 ± 0.0 ^a^
Am_SanluZhan7_	−161.1 ± 1.6 ^b^	2.2 ± 0.1 ^b^	−97.6 ± 0.1 ^b^	0.5 ± 0.0 ^c^
Am_Koshihikari_	−191.9 ± 5.9 ^c^	2.9 ± 0.0 ^c^	−99.1 ± 0.1 ^c^	0.8 ± 0.0 ^d^

The values are means ±SD, calculated from duplicate measurements; values with different letters in the same column are statistically different at *p* < 0.05.

**Table 4:** Textural properties (hardness and stickiness) of cooked rice and amylose gels.

Sample	Cooked Rice		Amylose Gel
	Hardness (N/10^4^)	Stickiness (N.s/10^2^)	Hardness (g/10^2^)
TaiZhou0206	1.4 ± 0.1	2.6 ± 0.4 ^d^	0.4 ± 0.1 ^b^
XiangGeng111	1.0 ± 0.0 ^e^	5.0 ± 1.0 ^e^	0.1 ± 0.0 ^a^
SanLuZhan7	1.5 ± 0.1 ^g^	0.6 ± 0.1 ^b^	1.3 ± 0.1 ^c^
Koshihikari	1.2 ± 0.1 ^f^	7.8 ± 0.4 ^f^	1.9 ± 0.1 ^d^

The values are means ± SD, calculated from duplicate measurements; values with different letters in the same column are statistically different at *p* < 0.05.

## Data Availability

Data is contained within the article or supplementary material.

## Supplementary Materials

The following supporting information can be downloaded at: https://www.mdpi.com/article/10.3390/foods11101516/s1, Figure S1: The CLDs and model fitting of amylose from rice TaiZhou0206 are given here as examples. a and b, model fitting of parent amylose and leached amylose respectively; Table S1: Starch molecular parameters of fully branched samples; Table S2: Amylose contents calculated from SEC data and starch molecular parameters of debranched samples; Table S3: Amylose contents measured by colorimetric method in isolated amylose samples; Table S4: Starch digestion parameters for isolated amylose; Interchain overlap.
