# Supplementary material for: Amylose Inter-Chain Entanglement and Inter-Chain Overlap Impact Rice Quality
Source: Foods. 2022 May 23;11(10):1516. doi: 10.3390/foods11101516 (PMC9141181; doi:10.3390/foods11101516)
Supplement: Supplementary file 1 [file foods-11-01516-s001.zip › foods-1709539-supplementary.pdf]

Supplementary information

Amylose inter-chain entanglement and inter-chain overlap impact rice quality

Figure S1. The CLDs and model fitting of amylose from rice TaiZhou0206 are given here as examples. a and b, model fitting of parent amylose and leached amylose respectively.

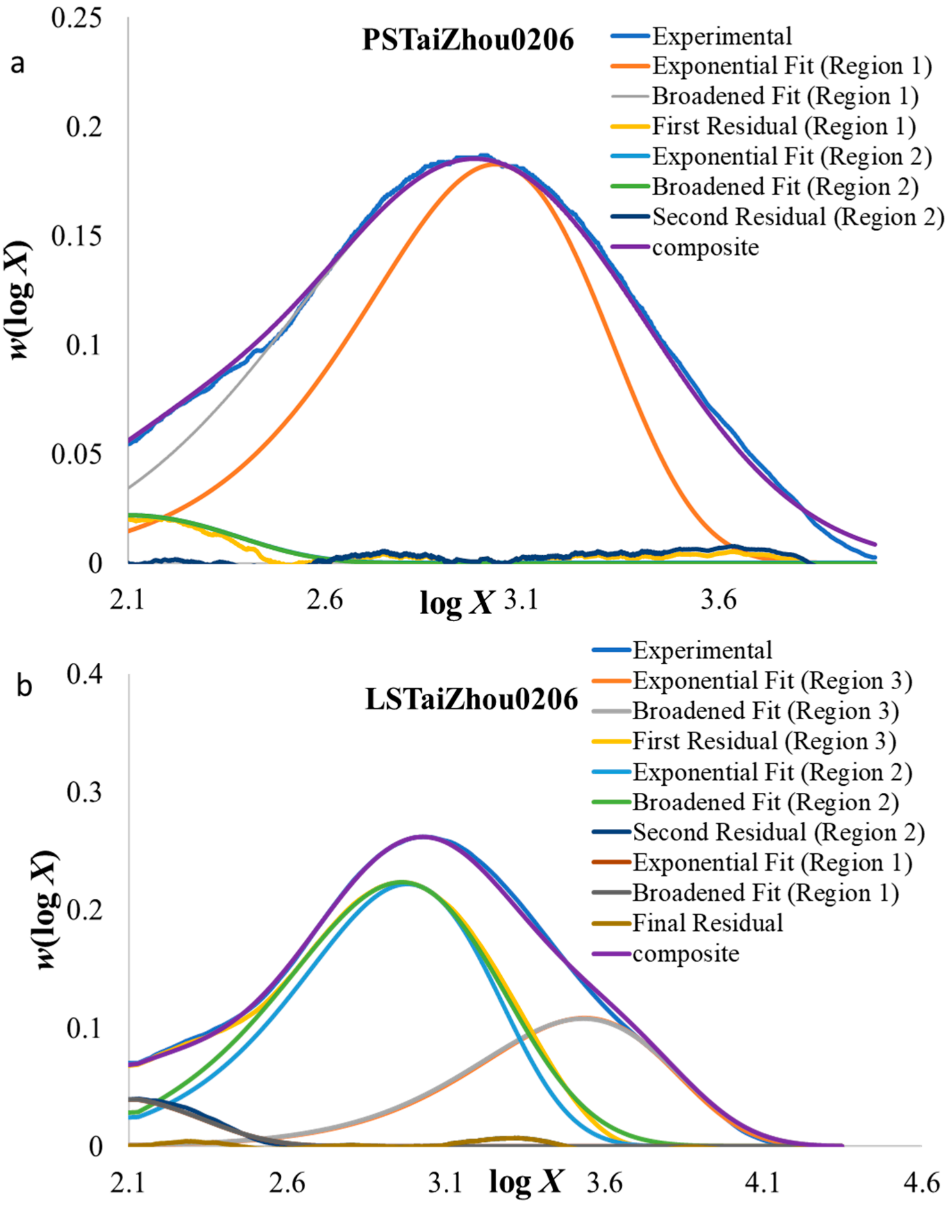

Table S1. Starch molecular parameters of fully branched samples.

Sample	$\bar{R}_{h,Ap}$	$\bar{R}_{h,Am}$	$\bar{R}_{h,whole\ starch}$
$PS_{TaiZhou0206}$	70.1 ± 2.9^{bcd}	13.0 ± 0.0^{f}	33.1 ± 1.1^{g}
$PS_{XiangGeng111}$	94.0 ± 3.0^{f}	12.7 ± 0.1^{e}	49.7 ± 1.0^{i}
$PS_{SanLuZhan7}$	75.6 ± 0.3^{de}	12.8 ± 0.1^{ef}	32.9 ± 0.1^{g}
$PS_{Koshihikari}$	79.9 ± 0.4^{e}	12.5 ± 0.0^{e}	39.0 ± 0.0^{h}
$LS_{TaiZhou0206}$	73.0 ± 1.6^{cd}	13.4 ± 0.1^{g}	19.7 ± 0.3^{e}
$LS_{XiangGeng111}$	92.1 ± 3.5^{f}	12.0 ± 0.1^{d}	22.9 ± 0.6^{f}
$LS_{SanLuZhan7}$	62.4 ± 0.6^{abc}	12.8 ± 0.0^{ef}	16.3 ± 0.1^{d}
$LS_{Koshihikari}$	93.1 ± 6.5^{f}	12.2 ± 0.1^{d}	22.6 ± 1.4^{de}
$Am_{TaiZhou0206}$	NA	8.5 ± 0.1^{c}	8.5 ± 0.1^{c}
$Am_{XiangGeng111}$	53.7 ± 1.1^{a}	7.8 ± 0.1^{a}	8.6 ± 0.0^{c}
$Am_{SanLuZhan7}$	62.0 ± 0.9^{ab}	8.1 ± 0.0^{b}	8.3 ± 0.0^{b}
$Am_{Koshihikari}$	NA	7.7 ± 0.0^{a}	7.7 ± 0.0^{a}

The values are mean ± SD, calculated from duplicate measurements; values with different letters for the same parameter are significantly different ($p < 0.05$).

Table S2. Amylose contents calculated from SEC data and starch molecular parameters of debranched samples.

Sample	Amylose content	$\bar{X}_{de,Am}$	$\bar{R}_{h,de,Am}$	$\bar{R}_{h,de,Ap}/\times100$
$PS_{TaiZhou0206}$	24.8±0.8^{c}	764±4^{f}	11.6±0.0^{f}	110.7±0.1^{a}
$PS_{XiangGeng111}$	10.5±0.7^{a}	681±19^{e}	10.8±0.2^{e}	112.2±0.0^{b}
$PS_{SanLuZhan7}$	32.5±0.2^{d}	500±1^{cd}	9.1±0.0^{cd}	110.9±0.0^{a}
$PS_{Koshihikari}$	13.4±0.0^{b}	713±15^{ef}	11.2±0.2^{ef}	111.1±0.0^{a}
$LS_{TaiZhou0206}$	31.7±0.6^{d}	874±36^{g}	12.4±0.3^{g}	112.7±0.1^{a}
$LS_{XiangGeng111}$	10.6±0.2^{a}	726±5^{ef}	10.9±0.0^{ef}	115.3±0.0^{c}
$LS_{SanLuZhan7}$	44.4±0.3^{e}	555±6^{d}	9.6±0.1^{d}	113.4±0.0^{b}
$LS_{Koshihikari}$	13.0±0.1^{b}	759±21^{f}	11.3±0.2^{ef}	113.4±0.0^{b}
$Am_{TaiZhou0206}$	80.2±2.4^{f}	479±27^{c}	8.9±0.2^{c}	170.0±0.5^{d}
$Am_{XiangGeng111}$	71.4±7.2^{f}	400±18^{b}	8.0±0.2^{b}	172.8±1.2^{d}
$Am_{SanLuZhan7}$	77.6±1.8^{f}	312±14^{a}	7.0±0.2^{a}	171.9±3.5^{d}
$Am_{Koshihikari}$	76.1±0.0^{f}	338±8^{ab}	7.2±0.1a	176.8±1.8^{d}

The values are mean ± SD, calculated from duplicate measurements; values with different letters for the same parameter are significantly different (p <0.05).

Table S3. Amylose contents measured by colorimetric method in isolated amylose samples.

Sample	Amylose content (%)
$Am_{TaiZhou0206}$	100.7±0.8[a]
$Am_{XiangGeng111}$	64.9±0.5[c]
$Am_{SanLuZhan\ 7}$	102.5±0.8[b]
$Am_{Koshihikari}$	101.2±0.1[ab]

The values are mean ± SD, calculated from duplicate measurements; Values with different letters for the same parameter are significantly different (p <0.05). The measurements obtained here are slightly above 100% because of the impurity of the amylose standard (Sigma A0512, Sigma-Aldrich Pty. Ltd.,Wisconsin, USA) used here. It was isolated from potato by butanol according to the specification.

Table S4. Starch digestion parameters for isolated amylose.

Sample	$k_1/10^{-2}$ min^{-1}	C_{res1} (%)	$k_2/10^{-2}$ min^{-1}	C_{res2} (%)
$Am_{Koshihikari}$	8.1±0.1^{a}	49.7±0.3^{d}	1.6±0.1^{a}	41.7±0.2^{d}
$Am_{TaiZhou0206}$	8.7±0.1^{dc}	34.8±0.4^{b}	1.8±0.1^{ab}	26.0±0.5^{b}
$Am_{SanLuZhan7}$	8.6±0.0^{bc}	41.8±0.6^{c}	1.7±0.1^{ab}	36.0±0.2^{c}
$Am_{XiangGeng111}$	8.9±0.0^{d}	32.3±0.7^{a}	1.9±0.1^{b}	24.6±1.1^{a}

The values are means ± SD, calculated from duplicate measurements, values with different letters in the same column are statistically different at p <0.05.

Interchain overlap

The extent of inter-chain overlaps varies with size and number concentration of the polymer. For simplicity, it is here assumed that the amylose molecule is linear (although in actuality it is slightly branched).

When a macromolecule dissoved in a solvent, the size of polymer random coil can be characterized by the radius of gyration (R_g). For a linear polymer, it is typically given by:

$R_g = a\, M^{0.6}$.

where M is the molar mass of the macromolecule and a is a constant. For a given total mass (m) of polymer, denote by n the number of chains per unit volume; V is total volume of polymer. If the molar mass of macromolecule is halved, the number of chains per unit volume doubles: $m = n_1 M_1 = n_2 M_2$.; $M_2 = M_1 / 2$, $n_2 = 2n_1$. So,

$$V_1 = n_1 \frac{4}{3}\pi R_1^{3} = n_1 \frac{4}{3}\pi ((aM_1)^{0.6})^{3} = \frac{4}{3} n_1 \pi a^{1.8} M_1^{1.8}$$

$$V_2 = \frac{4}{3} n_2 \pi a^{1.8} M_2^{1.8} = 2n_1 \frac{4}{3}\pi a^{1.8} (\frac{M_1}{2})^{1.8} = 2^{-0.8} \frac{4}{3} n_1 \pi a^{1.8} M_1^{1.8} \approx 0.57 V_1$$

When the concentration is above the critial concentration for inter-chain overlap, and the total mass is constant, the total occupied volume is reduced when molar mass of macromolecule is halved, resulting in less inter-chain ovelap.
